# Magnetic Biocatalysts of Pectinase: Synthesis by Macromolecular Cross-Linker for Application in Apple Juice Clarification

**DOI:** 10.17113/ftb.58.04.20.6737

**Published:** 2020-12

**Authors:** Marjan Nouri, Faramarz Khodaiyan

**Affiliations:** Bioprocessing and Biodetection Laboratory, Department of Food Science and Engineering, University of Tehran, Karaj, Iran

**Keywords:** magnetic microparticles, pectinase, chitosan, kefiran, apple juice

## Abstract

**Research background:**

Pectinase enzyme has become a valuable compound in beverage industry. One of the most significant concepts to overcome the drawbacks of using industrial enzymes is their immobilization. In the present study, magnetic chitosan microparticles were utilized as a substrate for pectinase immobilization. New methods of enzyme immobilization involve the use of non-chemical cross-linkers between the enzyme and the substrate. The aim of this study is to immobilize the pectinase enzyme using polyaldehyde kefiran as a macromolecular cross-linker on magnetic particles.

**Experimental approach:**

Pectinase was immobilized in four steps: relative oxidation of kefiran and its application as a cross-linker, production of magnetic iron(II) iron(III) oxide (Fe_3_O_4_) microparticles, coating of magnetic Fe_3_O_4_ microparticles with chitosan, and immobilization of the enzyme on the substrate, prepared by the use of oxidized kefiran cross-linker. Parameters such as cross-linking concentration, time and ratio of chitosan magnetic microparticles to enzyme were optimized. Fourier-transform infrared spectroscopy (FTIR), dynamic light scattering, transmission electron microscopy, and vibrating sample magnetometer were used to identify the groups and investigate the structures. The biochemical properties (stability of enzyme activity at different pH, temperature and time), enzyme reusability, kinetic parameters (*K*_m_ and *ν*_max_) and apple juice turbidity, using free and immobilized pectinase enzymes, were also measured.

**Results and conclusions:**

Cross-linker concentration, cross-linking time and the ratio of magnetic Fe_3_O_4_ microparticles with chitosan to enzyme were important factors in activity recovery of pectinase. FTIR analysis correctly identified functional groups in the structures. The results showed that after enzyme stabilization, the particle size and molecular mass, respectively, increased and decreased the magnetic saturation strength. According to the thermal kinetic study, the activity of the immobilized pectinase was higher than of its free form. The findings of this study indicate excellent stability and durability of the immobilized pectinase. Finally, a magnetic pectinase micro-biocatalyst was used to clarify apple juice, which reduced turbidity during processing.

**Novelty and scientific contribution:**

This study investigates the usage of kefiran oxidized as a new cross-linker for the immobilization of pectinase enzyme. Magnetic pectinase micro-biocatalyst has a good potential for industrial applications in the food industry, with high thermal stability.

## INTRODUCTION

In recent years, juice production technologies have attracted major attention due to the increased consumption of natural juices in order to improve their quality. Juices with added pectinase have a clearer appearance and filterability than enzyme-depleted counterparts (*1*). Freshly squeezed juice has a turbid appearance due to the colloidal dispersion of pectin (1.5 to 0.9%) in the cell wall structure, which is one of the most important disadvantages of fruit juice processing (*2*). Pectic substances are a complex of glycoside macromolecules with a high molecular mass. These substances can be found in plants and are responsible for the structure and integrity of plant tissue (*3*).

Several enzymes are used simultaneously to decompose pectic substances. Pectolytic enzymes or pectinases are a heterogeneous group of enzymes involved in the decomposition of pectic substances (*4*). The main industrial applications of pectinases include extraction, clarification and shredding of fruit and vegetable pulp (*5*). Commercial pectinases are usually a mixture of pectin-esterase, polygalacturonase, and pectate lyase, each affecting part of the pectin chain and converting it to soluble compounds (*6*, *7*). Pectinases as hydrolytic enzymes are used abundantly in different industries such as fruit juice extraction, coffee and tea fermentation, cotton scouring, water and wastewater treatment, and bleaching of paper (*8*).

The industrial enzymes and their applications are challenging due to restrictions associated with the production process or the type of enzyme used, substrate and product inhibitions, low operational stability, high production cost and difficult recovery (*9*). One of the most significant concepts to defeat these drawbacks is immobilization of the enzymes (*10*). Various benefits of immobilized enzymes include their enhanced enantioselectivity and reusability, improved operational stability and enzyme environment, easier reactor operation, suitable product separation, and their resistance to denaturation, degradation and aggregation (*9*, *11*). The improved stability and reusability can significantly reduce the cost of an enzyme and thus make the industrial application economically feasible (*12*). If an improper immobilization is used, immobilized enzymes usually demonstrate lower activity than the free enzymes. This phenomenon leads to partial blocking of the enzymes’ active sites, enhanced mass-transfer limitations between the enzymes and the substrate, and conformational changes in the enzymes (*9*). Therefore, the selection of an appropriate immobilization strategy depends on the physical and chemical characteristics of the enzyme and immobilization matrix (*10*).

Enzyme immobilization methods include surface adsorption to organic polymers, metal oxides and silica materials, entrapment in natural and synthetic polymers, ion exchange, cross-linking and covalent bonding to organic polymers (*11*). Enzyme immobilization in the composite structure has always been challenging. Until now, various substrates have been used to immobilize enzymes (*13*). These substrates should have the following basic properties: availability, tendency to bind to proteins, presence of free working groups and reactions with the target enzyme, hydrophilic strength, mechanical stability, tissue stiffness, flexibility in different geometrical structures, permeability, and appropriate surface for transfer, degradability, cost-effectiveness and safety of application (*14*, *15*).

In the present study, magnetic chitosan microparticles were used as a substrate for pectinase enzyme immobilization. Cationic biopolymer chitosan, as an available and non-toxic compound, is the most renewable biopolymer after cellulose (*16*). This biopolymer and its derivatives have great potentials for application in different fields, such as agriculture, food, textile, cosmetic and pharmaceutical industries, environmental protection and biomedical research (*17*). It is known that chitosan solubility in aqueous solutions is limited due to the presence of strong hydrogen bonds and intermolecular reactions. However, its solubility in acidic environments (organic, mineral and dilute acids) depends on crystallinity, polymerization, neutralization of amine groups, glucosamine distribution, solvent ionic strength, pH and concentration (*16*). Chitosan is formed by the protonation of amine groups of water-soluble salts in repeating units (*17*).

Core-shell structured magnetic composite particles possess unique physicochemical characteristics and shells which endow them with great application potentials in various fields (*18*). The benefits of these particles include their ability to assemble simple and rapid assembly, low cost, high loading capacity due to their large specific surface, presence of functional groups, and unlimited permeability in solutions (*18*). The utilization of magnetic particles for enzyme immobilization is a promising treatment strategy, as enzymes can be easily recovered by magnetic particles and recycled for further use (*19*). Previous studies have shown favourable results for the use of magnetic particles as a substrate for the immobilization of protease, catalase, phenylalanine ammonia lyase, lipase, β-glucosidase, peroxidase, pullulanase, α- and β-galactosidase, laccase, glucoamylase and invertase enzymes (*10*, *18*-*21*).

One method of enzyme stabilization is to apply cross-linkers and create a strong link between the enzyme and the substrate (*21*). Different cross-linking agents are known that can be utilized. Although glutaraldehyde remains cheap and versatile, some enzymes are inactivated by this reagent and its toxic nature. In fact, its small size enables it to reach the active site of the target enzyme, catalytically causing inactivation and accumulation of enzymes, thereby blocking their active sites which results in the loss of enzyme activity (*22*). Because of that, glutaraldehyde cannot be utilized widely in the food industry and its use is in contrast with the term green synthesis (*23*). In previous studies, polyaldehyde dextran, chitosan and pullulan were used as cross-linkers (*17*, *21*, *23*). Polysaccharides produced in kefir grains have a 1:1 glucose-to-galactose ratio, with a rotation angle of +68 (*24*). In the present study, oxidation-controlled kefiran was used as a non-chemical cross-linker. However, magnetic chitosan microparticles have not been investigated as substrates for pectinase immobilization by polyaldehyde kefiran as a cross-linker; therefore, further investigation is needed in this area. Kefiran is an extracellular polysaccharide with many hydroxyl groups that by partial oxidation provide the possibility of coupling between the enzyme and the carrier in a safe, non-toxic and efficient cross-linking (*25*).

**Fig. S1:**
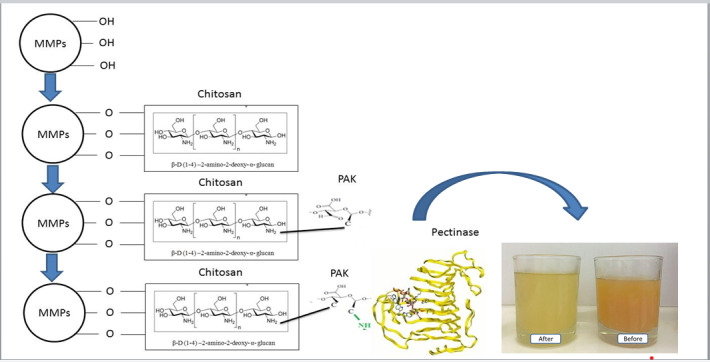
Scheme of enzyme immobilization steps

In the present study, pectinase was immobilized in four steps: (*i*) relative oxidation of kefiran and its application as a cross-linker, (*ii*) production of magnetic Fe_3_O_4_ microparticles, (*iii*) coating of magnetic Fe_3_O_4_ microparticles with chitosan, and (*iv*) immobilization of enzyme on the substrate, prepared by the use of oxidized kefiran cross-linker. Fig. S1 illustrated the steps of enzyme immobilization.

## MATERIALS AND METHODS

### Materials

Pectinase (lyophilized powder; EC 3.2.1.15) and chitosan (90.5% degree of deacetylation) were purchased from Sigma-Aldrich, Merck (Saint-Quentin-Fallavier, France). Kefir grains were obtained from a household in Tehran, Iran. In addition, iron(II) chloride tetrahydrate (FeCl_2_·4H_2_O, 99.7%), iron(III) chloride hexahydrate (FeCl_3_·6H_2_O, 99%), hydrochloric acid (HCl), sodium hydroxide (NaOH), sodium periodate (NalO_4_), 2,4-dinitrophenylhydrazine (DNPH), apple pectin (100 molecular mass, 120 kDa, 85 to 90% degree of esterification, 65% galacturonic acid), and 3,5-dinitrosalicylic acid (DNSA) were acquired from Sigma-Aldrich Chemical Co., Merck (Saint Louis, MO, USA). All aqueous solutions were prepared with deionized water that had been passed through a Milli-Q Plus water purification system (Millipore, Bedford, MA, USA). All other chemicals were of analytical grade and used without purification.

### Macromolecular cross-linking of polyaldehyde kefiran

For partial oxidation of kefiran, sodium phosphate-buffered (SPB) NalO_4_ (*c*(NalO_4_)=100 to 600 mM) was added to the polysaccharide kefiran as an oxidizing solution. Kefiran was kept and oxidized in a dark place for 30 to 150 min. After oxidation, an ethylene glycol solution (0.3 mL) was added to terminate oxidation, and the polyaldehyde kefiran solution was kept at 4 °C overnight (*26*).

### Determination of the oxidation rate of polyaldehyde kefiran

In this experiment, oxidized kefiran (0.3%, 3·10^−4^ g/L) was added to a freshly prepared solution of DNPH (10 mL, 1% *m/V*). The mixture was kept at ambient temperature for 1 h and then centrifuged (Universal 320; Hettich, Tuttlingen, Germany) at 8000×*g* for 10 min. The unreacted DNPH in the supernatant was measured using an ultraviolet spectrophotometer (Thermo Scientific, Madison, WI, USA) at 357 nm and calculated as follows (*27*):


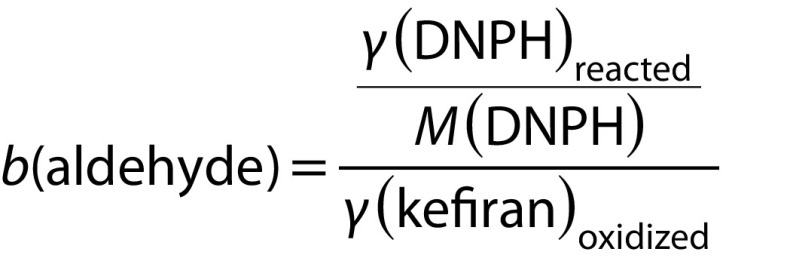


where *M*(DNPH)=198.14 g/mol, *γ*(DNPH)_reacted_ is in mg/L and *γ*(kefiran)_oxidized_ is in g/L. The oxidized kefiran was used as a macromolecular cross-linker. The oxidation rate of hydroxyl groups was calculated according to the method developed by Väisänen *et al*. (*28*).

### Synthesis of magnetic chitosan-Fe_3_O_4_ microparticles

First, 2 g of FeCl_3_ were mixed with 1 g of FeCl_2_ at a ratio of 2:2 after dissolving in hydrochloric acid (0.5 mL). Next, the aqueous solution of FeCl_3_ (45 mL) and FeCl_2_ (45 mL) was added to a container with 80 mL of distilled water under nitrogen atmosphere. After stirring at 100×*g* and adding 50 mL of 1.5 M NaOH, the solution reached pH=10, and a black precipitate was produced. Next, iron oxide particles were removed from the medium using a magnet. In order to remove the additives, the particles were washed twice with distilled water and once with ethanol. At the end of this step, magnetic Fe_3_O_4_ microparticles were obtained. To cover 200 mL of microparticle suspension (0.25%, *m/V*), 1% chitosan solution and 45 mL of 1% acetic acid were added, and the medium was completely sealed. Afterwards, Fe_3_O_4_ microparticles were coated with chitosan at (40±3) °C for 2 h under constant stirring (200×*g*) by overhead laboratory stirrer. The final magnetic product was separated by a permanent magnet and washed twice with distilled water. The final product was dried by freeze-drying and kept in dark. The magnetic Fe_3_O_4_ microparticles with chitosan were dried overnight at room temperature and stored at low temperature for subsequent experiments (*21*, *29*).

### Immobilization of pectinase onto the carrier

In the green synthesis, polyaldehyde kefiran was utilized as a cross-linker rather than chemical cross-linkers comprising reactive aldehyde (-CHO) groups in the molecule. The aldehyde groups react with amine (-NH_2_) groups to synthesise a Schiff base ((-CH=N-) and this process was utilized by chitosan functional amine groups to cross-link with polyaldehyde kefiran groups (*29*). Magnetic chitosan microparticles were mixed (175×*g*) with pectinase (10 mg protein) in SPB (100 mM, pH=6) at a ratio of 1:1 to 1:5 (*m/m*) and kept for 30 min. Polyaldehyde kefiran cross-linker (*φ*=4 to 0.5% of total solution) was prepared, and the mixture was incubated for 3 to 24 h. Next, sodium tetrahydridoborate (5 mg) was added, and the mixture was kept for 30 min to terminate the process. The immobilized pectinase was magnetically separated, washed with buffer, and enzyme activity was evaluated. It was stored at 4 °C until use (*30*). The enzyme activity recovery is defined as the ratio of immobilized enzyme activity to total free enzyme activity at the beginning of the reaction (*31*):


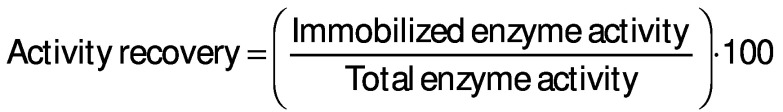


### Investigation of the structural and physicochemical properties of magnetic microparticles with and without chitosan, polyaldehyde kefiran and pectinase

To confirm the functionality of the magnetic microparticles, Fourier-transform infrared spectroscopy (FTIR) was applied using an FTIR spectrophotometer (Cary 660 series; Agilent Technologies, Victoria, Australia), equipped with a deuterated triglycine sulfate detector in the scanning range of 400 to 4000 cm^-1^. FTIR provides an experimental and interpretive framework of the structure, interactions of natural polymer systems and physical characteristics (*31*, *32*). The surface charge and hydrodynamic size of the particles was measured using dynamic light scattering instrument (Malvern Instruments Ltd., Worcestershire, UK). The volume size distribution was calculated from the intensity of the light diffracted at each angle. Transmission electron microscopy (TEM; JEOL, Leoben, Austria) was also used to acquire images that show the surface morphology and structure of the particle solution. The effect of magnetic susceptibility and coating on the paramagnetic properties of synthesized particles was determined using vibrating sample magnetometer at room temperature (VSM, Kashan, Iran).

### Evaluation of pectinase efficacy

Pectinase (0.5 mL) was incubated with 1% apple pectin solution (prepared in phosphate buffer; pH=6, 100 mM) for 20 min at (50±2) °C. Next, DNSA reagent (2 mL) was added to the mixture, boiled for 15 min, and then cooled. The absorbance of the mixture was measured at 575 nm with an ultraviolet spectrophotometer (Thermo Scientific). The enzyme activity was determined as the amount of enzyme required to release 1 mol of β-galacturonic acid per min under optimal conditions ((50±2) °C, pH=6). The protein concentration in the supernatant was measured using Bradford reagent with standard bovine serum albumin (1 mg/mL) (*26*).

### Investigation of biochemical properties of free and immobilized pectinase

Biochemical properties of pectinase were determined by investigation of the stability of enzyme activity under different acidic, alkaline and temperature conditions by incubating a certain amount of free and immobilized enzymes under variable pH values (pH=2 to 8) at constant temperature (20 to 80 °C) or different temperatures with constant pH. Also, enzyme activity was measured within five-day intervals under standard conditions at constant pH=4 and temperature (20 °C) for a shelf-life of 30 days (*6*).

### Reusability of immobilized pectinase

Reusability of immobilized pectinase was measured by reducing its activity after each application in the reaction medium for substrate hydrolysis. At the end of each step, the enzyme was separated from the reaction medium using a magnet and washed with phosphate buffer (100 mM, pH=6.0). Then, this enzyme was resuspended in substrate solution to measure its activity and the process was repeated in 10 cycles (*N*=10). Residual activity was measured after each cycle (100% residual activity after the first cycle) (*32*).

### Kinetics of the free and immobilized pectinase activity

The kinetic parameters for both free and immobilized pectinases were evaluated using the Michaelis-Menten and Lineweaver-Burk plots. These were carried out by plotting different concentrations of pectin solution (2.0 to 8.0 mg/mL) against reaction rate:


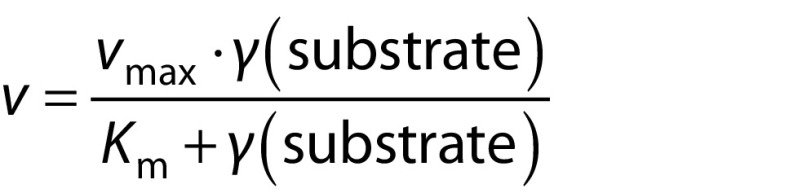


where *γ*(substrate) is the substrate concentration (mg/mL), *v*_max_ is the maximum reaction rate obtained at an infinite substrate concentration (μmol of galacturonic acid/per min), and *K*_m_ is the constant rate (mg/mL) (*6*).

### Application of immobilized pectinase in apple juice clarification

The potential of immobilized pectinase for clarification of apple (var. Golab) juice was examined. Fresh apple juice was centrifuged (Universal 320; Hettich) at 5000×*g* for 20 min, and the supernatant was used for the clarification process. The immobilized pectinase (with an equivalent amount to free pectinase) was mixed with juice diluted with water (*φ*(juice)=20%) and treated for 150 min at 50 °C. After enzymatic treatment, juice clarity was determined as turbidity, using a spectrophotometric (model CE2502 BIOQUEST; CECIL Instruments, Cambridge, UK) method (*26*).

### Statistical analysis

Data presented in various studies were plotted using OriginPro software v. 9.4 (*33*) and expressed as mean value±standard error. Each value represents the mean of three independent experiments, with an average standard deviation of <5% (*34*).

## RESULTS AND DISCUSSION

### Results of controlled oxidation of kefiran

**Fig. 1:**
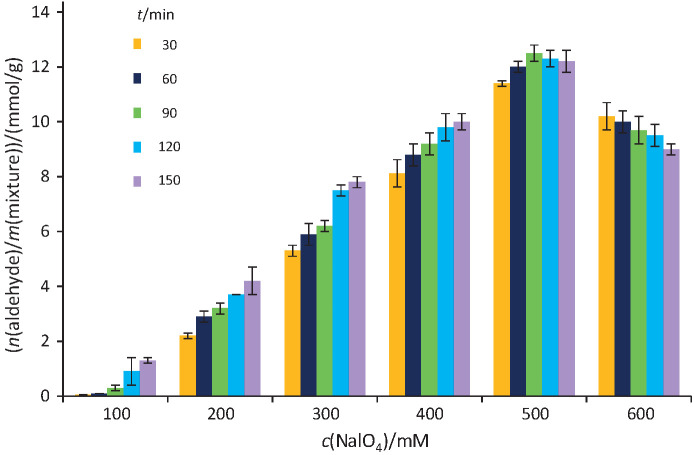
Aldehyde content generated in kefiran after oxidation with sodium periodate (100–600 mM) determined by 2,4-dinitrophenylhydrazine (DNPH) method. The measurements were performed in triplicate and the error bar represents the percentage error

The kefiran cross-linking macromolecule was prepared by controlled oxidation with sodium periodate. Periodic acid and its salts can specifically oxidize kefiran. The rate of oxidation to produce the maximum amount of aldehyde group depends on the concentration of NalO_4_ and reaction time. Therefore, the effects of NalO_4_ concentration and reaction time on the produced aldehyde groups were investigated based on the dinitrophenylhydrazine test (Fig. 1).

An increase in the concentration of NalO_4_ increased the concentration of aldehyde group to a maximum of 500 mM within 120 min. However, at concentrations above 500 mM, the amount of polyaldehyde kefiran decreased. Kefiran oxidation may be inhibited at higher concentrations of NalO_4_. Also, longer reaction time did not increase the oxidation significantly; in other words, the results of oxidation after 120 and 150 min differ slightly. Finally, the oxidized kefiran (12.5 mmol/g) was dissolved in SPB (100 mM, pH=6) and used as the polyaldehyde kefiran cross-linking macromolecule. According to the method described previously (*21*), the oxidation rate of hydroxyl groups was 35.71% in the present study.

### Immobilization of pectinase onto polyaldehyde kefiran and chitosan magnetic microparticles with oxidized kefiran as cross-linker

Synthetic cross-linkers such as glutaraldehyde and carbiodiimide were used in the past. Nowadays, they are used less because of their small size and toxicity. They are also considered harmful to living organisms because of their toxic properties; therefore, they cannot be used in the food industry. In recent years, many authors have applied dextran, xanthan, pectin and dextran aldehyde as natural cross-linkers instead of the synthetic ones and reported positive results (*23*).

The aim of the present study was to immobilize pectinase using polyaldehyde kefiran as a macromolecular cross-linker on magnetic particles. Reactive aldehyde groups of oxidized kefiran can form amino groups in lysine or hydroxyl lysine of the enzyme, as well as chitosan group in the Schiff base, which in turn prevents enzyme removal during the reaction. The crosslinking parameters (concentration and time of cross-linking) have direct effects on enzyme loading, activity recovery and operational stability of the prepared magnetic biocatalyst. Overall, to optimize the rate of cross-linking between the enzyme and magnetic particles, maximum recovery of enzyme activity is required (*35*).

**Fig. 2:**
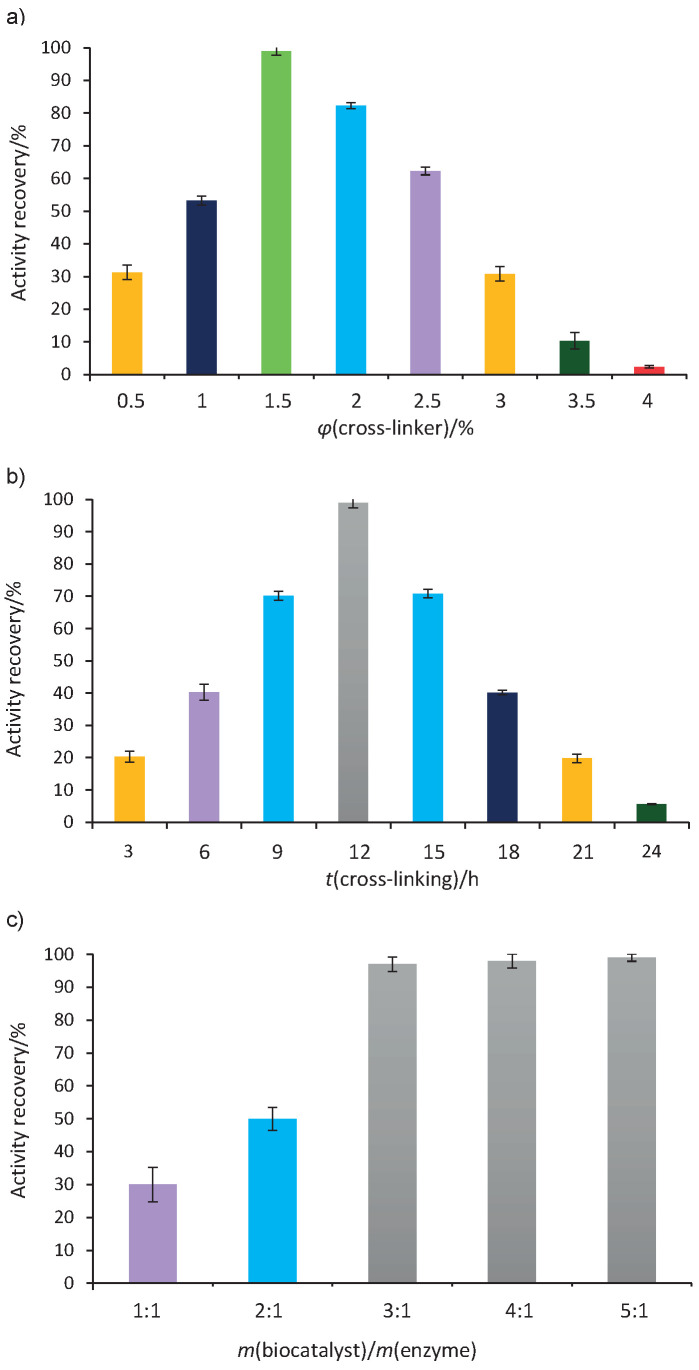
Effect of: a) cross-linker concentration, b) cross-linking time and c) ratio of magnetic Fe_3_O_4_ microparticles with chitosan (biocatalyst) to enzyme (protein) on pectinase activity recovery. The measurements were performed in triplicate and the error bar represents the percentage error

The effect of cross-linker volume fraction (*φ*) on the recovery of pectinase activity in the immobilized form was investigated by varying the volume fraction from 0.5 to 4% (Fig. 2a). The published results showed that the amounts of cross-linkers had remarkable effects on the activity recovery (*36*). Low volume fraction of cross-linker (0.5%) caused the decrease of enzyme activity recovery due to reduced enzyme cross-linking. In fact, low volume fractions of cross-linker do not have the required capacity to bind Schiff bases (covalent bond) and efficiency of enzyme cross-linking immobilization is low, while it is improved by increasing the volume fraction of cross-linker. In the present study, maximum activity recovery was observed with magnetic Fe_3_O_4_ microparticles and 1.5% polyaldehyde kefiran. In addition, an increase in the cross-linker volume fraction resulted in enzyme reorganization, denaturation and subsequent activity loss. Previous reports showed that the excess of glutaraldehyde caused the formation of clusters with mass transfer limitations, which resulted in lower activity recovery (*37*, *38*).

Cross-linking time is another important factor in enzyme immobilization (*31*). In this study, as the cross-linking time advanced, the recovery of enzyme activity increased (Fig. 2b). Maximum activity recovery of pectinase immobilized on magnetic Fe_3_O_4_-chitosan microparticles has been observed within 12 h of linking with polyaldehyde kefiran. Further increase in the cross-linking time resulted in a gradual decrease in pectinase activity recovery due to excessive cross-linking, which led to the chemical modification of enzyme (*39*). Also, prolonged cross-linking time could restrict the enzyme flexibility and disable its activity by blocking the enzyme active sites (*38*).

To maximize pectinase immobilization, the ratio of chitosan magnetic particles to enzyme (1:1 to 5:1) was optimized (Fig. 2c). At the lowest ratio, the amount of magnetic Fe_3_O_4_ microparticles with chitosan was insufficient for pectinase loading, leading to its decreased activity. As the ratio increased, the activity recovery improved significantly, depending on the type of microparticle. In magnetic Fe_3_O_4_ microparticles with chitosan, the particle-to-enzyme ratio of 3:1 had the highest pectinase activity recovery. However, as the ratio of magnetic Fe_3_O_4_ microparticles with chitosan to enzyme increased further, the activity recovery remained constant, possibly due to maximum enzyme loading onto the carrier (*4*). In the present study, the effect of the increase of the values of these three factors on the rate of activity recovery is in line with the results of some researchers (*26*, *36*, *38*).

### Formation of magnetic Fe_3_O_4_ microparticles with or without chitosan, and the complex containing magnetic microparticles, chitosan, oxidized kefiran and pectinase

**Fig. 3:**
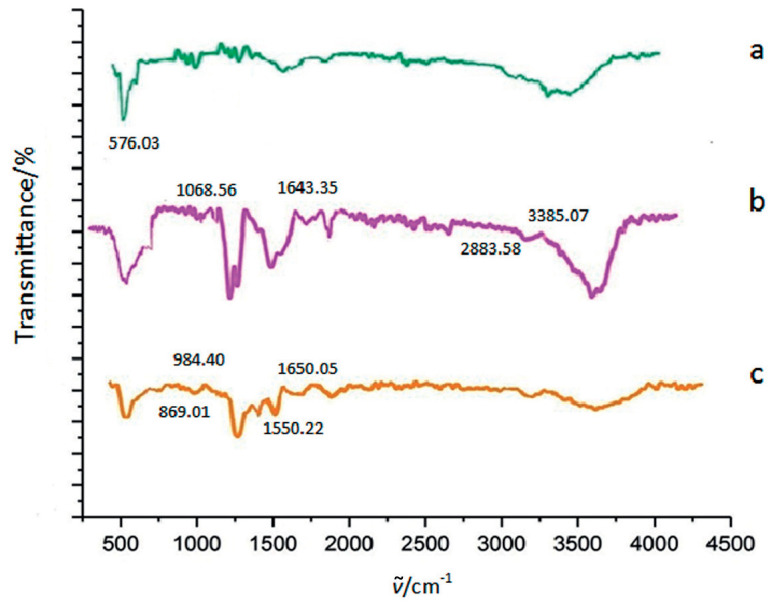
FTIR spectra of the magnetic Fe_3_O_4_ microparticles (a), magnetic Fe_3_O_4_ microparticles with chitosan (b), and the complex containing magnetic microparticles, chitosan, oxidized kefiran and pectinase (c)

The structure of magnetic Fe_3_O_4_ microparticles with and without chitosan, and the complex containing magnetic microparticles, chitosan, oxidized kefiran and pectinase was determined using FTIR in the *ṽ* range of 4500 to 500 cm^-1^ (Fig. 3).

The specific peak at 576.03 cm^−1^ was related to the intrinsic tensile vibrations of Fe–O in the Fe_3_O_4_ structure (spectrum a in Fig. 3). This peak was observed in all structures (a, b and c).

When chitosan was used as a coating for microparticles, peaks similar to native chitosan were observed. The peaks at nearly 3385.07 cm^-1^ were related to tensile vibrations O–H and NH (-NH_2_), and peaks at nearly 1068.56 cm^−1^ indicated N-H bend of R-NH_2_ and C–O–C. In addition, the peaks at 2883.58 and 1643.35 cm^−1^ (observable in spectra a and b in Fig. 3) were attributed to the tensile vibrations of C–H and N–H, respectively (spectrum b in Fig. 3), which confirmed the successful preparation of magnetic Fe_3_O_4_ microparticles with chitosan (*40*, *41*).

The structure of the final complex containing magnetic microparticles, chitosan, oxidized kefiran and pectinase is shown (spectrum c in Fig. 3). After enzyme immobilization, the peaks in spectra a and b are still present, but some peaks are slightly altered or overlapped due to enzyme loading and the presence of new linker groups. The peaks at 869.01, 892.11, 927.37 and 984.40 cm^−1^ indicated the presence of glucose, galactose, and beta cross-linker in the structure of polyaldehyde kefiran polymer, respectively (*25*). Also, the peak at 576.02 cm^−1^ was related to the Fe_3_O_4_ structure. In addition to the peaks associated with the structure of kefiran polymer, chitosan and iron oxide peaks in the range of 1550.22 to 1650.05 cm^−1^ indicated the presence of amino acids in pectinase enzyme (the complex containing magnetic microparticles, chitosan, oxidized kefiran and pectinase).

**Fig. 4:**
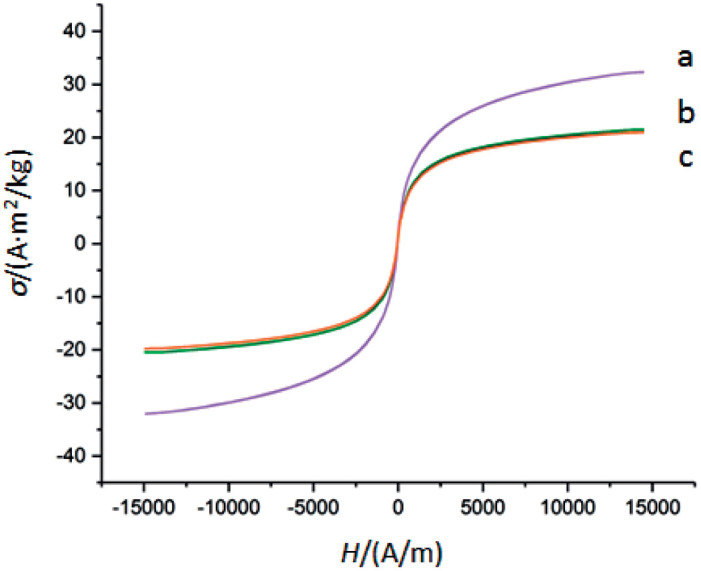
Magnetic hysteresis loops (magnetic saturation *σ* vs. magnetic field strength *H*) of: magnetic Fe_3_O_4_ microparticles (a), magnetic Fe_3_O_4_ microparticles with chitosan (b), and the complex containing magnetic microparticles, chitosan, oxidized kefiran and pectinase (c)

The magnetic saturation (*σ*) for magnetic Fe_3_O_4_ microparticles, magnetic Fe_3_O_4_ microparticles with chitosan, and the complex containing magnetic microparticles, chitosan, oxidized kefiran and pectinase was 32.70, 19.90, and 20.15 (A·m^2^)/kg, respectively (curves a, b and c, respectively, in Fig. 4). The two diagrams were roughly uniform, but could be deduced from the hysteresis rings. Overall, high magnetic saturation indicated high magnetizability, which suggests adequate isolation with conventional magnets in the present study. The results showed that after enzyme stabilization, the particle size and molecular mass, respectively, increased and decreased the magnetic saturation strength. In other words, the higher the particle mass, the lower the magnetic saturation strength, which is in line with previous research (*42*).

Since residual magnetization and forcing magnets were close to zero for almost all three samples, they exhibited behaviours similar to superparamagnetic particles and showed adequate capacity to disperse after the magnetic field is removed. Magnetic particles can easily separate the highly magnetized particles of immobilized enzyme (*43*).

**Fig. 5:**
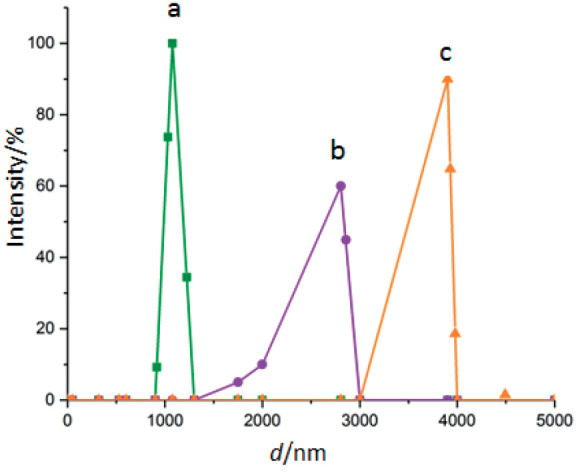
Dynamic light scattering curves of: magnetic Fe_3_O_4_ microparticles (a), magnetic Fe_3_O_4_ microparticles with chitosan (b), and the complex containing magnetic microparticles, chitosan, oxidized kefiran and pectinase (c)

The results of particle size (*d*) analysis showed that the particle size of magnetic Fe_3_O_4_ microparticles was about 1 μm in the first step, which increased to almost 4 μm in the final step (curve a in Fig. 5). This is attributed to the large particle size from the beginning of the process (curve b in Fig. 5), which increased by adding each coating to the primary microparticles (curve c in Fig. 5).

**Fig. 6:**
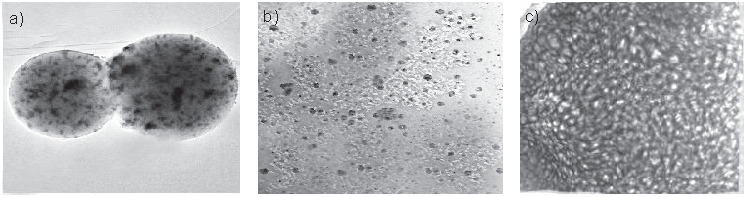
Transmission electron microscopy images of: a) magnetic Fe_3_O_4_ microparticles, b) magnetic Fe_3_O_4_ microparticles with chitosan, and c) the complex containing magnetic microparticles, chitosan, oxidized kefiran and pectinase

The TEM results of the samples of magnetic Fe_3_O_4_ microparticles (Fig. 6a) showed that their spherical structure is almost regular and coherent, with a smooth surface. In the samples of the complex containing magnetic microparticles, chitosan, oxidized kefiran and pectinase (Fig. 6b), chitosan was well-seated on the particles. Increase of the particle size of the complex (2805 to 3904 nm) indicates the enzyme linking to particles (Fig. 6c). Also, rough surfaces caused by the magnetic properties and size of particles were observed.

### Biochemical properties of the immobilized enzyme (temperature, time and pH stability)

**Fig. 7:**
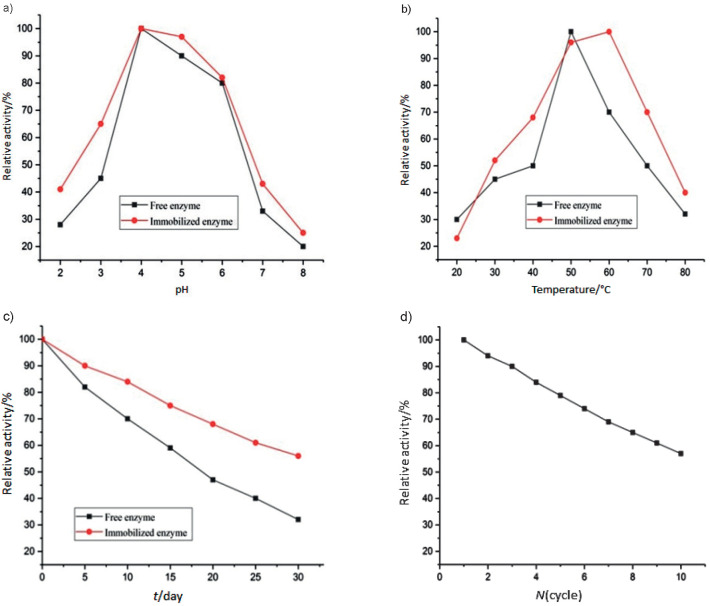
Influence of different parameters on relative pectinase activity (free and immobilized on magnetic Fe_3_O_4_ microparticles with chitosan): a) pH values (pH=2–8) at constant temperature (40 °C), b) temperature (20-80 °C) at constant pH=4, c) stability with time (0-30 days), and d) reusability of the complex containing magnetic microparticles, chitosan, oxidized kefiran and pectinase during 1-10 cycles

The relative stability of free and immobilized pectinase on magnetic Fe_3_O_4_ microparticles with chitosan at different pH (pH=2 to 8) is shown in Fig. 7a. The optimal pH was pH=4 for free and immobilized enzymes because at that value the highest enzyme relative activities were obtained. At the highest pH (*i.e.* pH=8), free and immobilized pectinase activities were 20.06 and 25.51%, respectively. The enzyme immobilized on magnetic Fe_3_O_4_ microparticles had a significantly higher relative activity than the free enzyme. Overall, magnetic particle substrates facilitate pectinase activity. Also, the enzyme disruption decreases at optimal pH conditions, which consistent with the results reported previously (*6*).

Stability of the relative activity of free and immobilized pectinase on magnetic Fe_3_O_4_ microparticles with chitosan at different temperatures is shown in Fig. 7b. The optimal temperature for free and immobilized enzymes on was 50 and 60 °C, respectively. At the highest temperature (80 °C), activities of free and immobilized enzymes were 32.1 and 40.1%, respectively. According to the present results, at high temperatures (*e.g*. 80 °C), free and immobilized enzymes showed insignificant differences. On the other hand, significant differences were observed between the samples from 30 to 70 °C, and immobilized enzymes exhibited higher thermal resistance. Moreover, the optimal temperature for immobilized enzyme activity increased by 10 °C, that is, it shifted from 50 to 60 °C, and the enzyme showed resistance to denaturation. However, at temperatures above 60 °C, the links between the cross-linker, enzyme and magnetic particles were probably broken, and the relative activity of the target enzyme reduced (*6*).

Another reason for the increased thermal stability of the immobilized enzyme is water depletion in the immobilized phase, which plays an important role in hydrophobic reactions, polarity reduction, and hydrophobic accumulation of proteins in the aqueous solution (*44*). The rate of enzyme loading and linkage in the microparticles was not very high. Therefore, the heat resistance was investigated because the particles are more closely linked to each other at high temperatures, filling the active sites of enzyme and reducing its activity. The rate of heat resistance of β-galactosidase immobilized on the microparticles was determined (*45*).

Furthermore, the storage stability of free and immobilized enzymes was determined within 5-day intervals for 30 days. The results showed that after a month, the residual activity of free and immobilized pectinase was 32.11 and 56.47%, respectively (Fig. 7c). Appropriate stability of immobilized pectinase during storage may be due to cross-linking with polyaldehyde kefiran on chitosan magnetic particles, which prevents possible accumulation at the active sites of enzyme (*43*).

### Reusability of enzyme

The diagram of the relative activity of the immobilized pectinase enzyme on magnetic Fe_3_O_4_ microparticles with chitosan with repeated applications is shown in Fig. 7d. Generally, enzymes are widely used in the food industry because of their activity as catalysts; however, they are expensive believing their reuse. Therefore, new methods of enzyme immobilization have been suggested for the reuse of enzymes. In the present study, removal of the immobilized pectinase enzyme on magnetic chitosan particles was investigated after catalytic activity and reuse of the enzyme ten times.

Determination of the number of reusable loads per enzyme is a key factor in cost-effective application of immobilized enzymes (*33*). However, reduced enzyme activity after repeated use is related to enzyme denaturation or loss of enzymatic links and cross-linkers (*6*). The decrease in the relative activity of enzyme after reuse is probably due to the release and separation of pectinase from the substrate by magnetic chitosan, which occurs due to separation and washing in each step; however, reduction of relative activity of pectinase in magnetic Fe_3_O_4_ microparticles with chitosan after ten repeated applications was 57.11%. The results showed that magnetic particles were more successful in maintaining this property.

### Kinetic parameters of free and immobilized enzymes

The kinetic parameters demonstrated that the *K*_m_ of the free enzyme (3.201 mg/mL) was higher than of the immobilized enzyme (2.713 mg/mL). The enzyme remained flexible even after cross-linking so it was still able to connect to the substrate (*6*, *23*, *46*).

A decrease in *ν*_max_ for free pectinase (0.595 μmol of galacturonic acid per min) compared with the *ν*_max_ for immobilized pectinase (0.924 μmol of galacturonic acid per min) showed that the rate of pectin hydrolysis increased after immobilization (*6*). The existence of covalent cross-linking between polyaldehyde kefiran and pectinase inhibits the aggregation of molecular mass due to mass transfer from the matrix surface (*23*, *46*).

### Clarification of apple juice by the complex containing magnetic microparticles, chitosan, oxidized kefiran and pectinase

The turbid appearance of fruit juice is attributed to the presence of pectic components. Accordingly, breakdown of these polysaccharides by pectinase can improve the quality and storage stability of apple juice (*47*). In the current study, apple (var. Golab) juice was treated with immobilized and free pectinase. Juice clarification was measured in terms of turbidity reduction, using the spectroscopic method (Table 1).

**Table 1:** Clarification of apple juices by free and immobilized pectinase

*t*/min	Turbidity/%	
Free pectinase	Immobilized pectinase
0	100.0±1.1	100.0±1.3
30	87.3±2.6	90.1±2.4
60	81.0±3.0	85.4±1.8
90	76.2±2.1	79.2±2.8
120	70.1±3.1	75.0±1.5

At the beginning of clarification, free pectinase reduced the rate of turbidity from 100 to 87.3%, whereas the immobilized pectinase enzyme reduced the turbidity from 100 to 90.1% within 30 min. As time progressed, free pectinase treatment reduced the turbidity of apple juice up to 70.1%, while immobilized pectinase treatment reduced turbidity to 75% after 120 min of treatment.

## CONCLUSIONS

The results of the present study showed the potential use of polyaldehyde kefiran as a cross-linking agent for immobilization of pectinase on magnetic Fe_3_O_4_ microparticles with chitosan. Our findings showed that the most appropriate rate of polyaldehyde kefiran cross-linker was 1.5% within 12 h of contact time; the highest recovery rate of pectinase activity was observed at magnetic Fe_3_O_4_ microparticles with chitosan-to-enzyme ratio of 3:1. Particle size ranged from 1000 (magnetic Fe_3_O_4_ microparticles) to 4000 nm (the complex containing magnetic microparticles, chitosan, oxidized kefiran and pectinase). The results of pectinase immobilization showed improvements in the biochemical properties (*i.e.* temperature, time and pH stability) and apple juice clarification parameters. It can be concluded that magnetic pectinase micro-biocatalyst, with high thermal stability, has a good potential for industrial applications in food industry.
